# Exploring Multidrug-Resistant Gram-Positive Bacteria on Electronic Devices: A Comparative Experimental Study Based on the Gender of Healthcare and Nonhealthcare Workers

**DOI:** 10.1155/cjid/8520827

**Published:** 2025-10-06

**Authors:** Mawahib Ahmed, Shahad Nasser, Basmah Alharbi, Mohsina Huq, Amal Hussain, Amal Mackawy, Mishaal Alrasheed, Afshan Zeeshan Wasti

**Affiliations:** ^1^Department of Medical Laboratories, College of Applied Medical Sciences, Qassim University, Buraydah 51452, Saudi Arabia; ^2^Department of Basic Health Sciences, College of Applied Medical Sciences, Qassim University, Buraydah 51452, Saudi Arabia; ^3^Department of Laboratory and Blood Bank, King Fahd Specialist Hospital, Buraydah 52211, Saudi Arabia

**Keywords:** antibacterial resistance, bacteria, healthcare professional, microbial contamination, mobile phone screen, pathogenic bacteria

## Abstract

**Background:**

Electronic device screens can harbor harmful microbes, potentially facilitating the transmission of diseases when users interact with these surfaces. This study aimed to investigate the presence of gram-positive bacterial contamination and identify potentially harmful bacteria in these screens. A total of 200 samples were collected from the screens of devices of healthcare and nonhealthcare workers. Cotton swabs collected samples from device screens, including cellphones and iPads/PCs. These samples were cultured and tested to identify bacterial organisms and then assessed for antibiotic resistance using the disc diffusion method. Statistical analysis was performed using the *t*-test and ANOVA to compare the data obtained from the two groups.

**Results:**

This study found 12 types of bacteria on device screens used by healthcare and nonhealthcare workers, irrespective of sex. *Staphylococcus epidermidis* was the most frequently detected bacterium in both groups. However, *Staphylococcus aureus* and *Staphylococcus anaerobes* were present in males but absent in females. Devices belonging to male participants exhibited statistically higher levels of bacterial contamination compared to those of the female participants (*p* < 0.05). Cell phones showed greater bacterial contamination than iPads/PCs. While six bacterial isolates exhibited resistance to antibiotics, *Staphylococcus sciuri*, *S. aureus*, *Staphylococcus hominis subspecies hominis*, and *S. epidermidis* showed multidrug resistance (MDR).

**Conclusion:**

This research found that all the tested mobile phones harbored microorganisms, indicating they could transmit diseases. This emphasizes the critical need for hand hygiene following use to curtail the propagation of infection and bolster overall public health outcomes.

## 1. Introduction

Bacterial contamination on the screens of electronic devices such as mobile phones and iPads poses a potential health risk, as these devices are frequently used in everyday life. Bacteria can be particularly harmful to health, especially in people with chronic health problems [1].

Devices can be contaminated with bacteria to varying degrees, depending on their use and the environment. Studies have shown that mobile phones and other handheld devices can pick up bacteria from food, skin, or respiratory droplets [2]. A study conducted in large cities that focused on mobile phone subscribers found that approximately half of the mobile phones contained bacteria. Notably, a significant proportion of more than a third of the phones examined contained dangerous levels of potentially pathogenic organisms [3]. This is alarming, as people outside the hospital may not have the same awareness of equipment hygiene as healthcare workers (HCWs), increasing the likelihood of bacterial presence and transmission to new surfaces [4].

The impact of bacterial contamination of devices is not only limited to the well-being of the user but also poses a threat to the public health. Such devices can spread infections, especially in communal situations such as schools, offices, or when traveling [5]. In particular, shared devices such as tablets or computers used in educational institutions are likely to be contaminated with microbes, which could lead to disease outbreaks, such as influenza and gastroenteritis [6]. This emphasizes the need to develop appropriate education and management strategies to prevent contamination of devices by non- HCWs (NHCWs) [2].

Common infectious bacteria most commonly spread by devices include *Escherichia coli* and *Bacillus*, which cause infections such as urinary tract infections (UTIs), *Staphylococcus* species that cause acne infection, and *Streptococcus*. In addition, these devices can cause surgical or wound site infections, pneumonia (caused by *Klebsiella* and *Acinetobacter* species), bloodstream infections, and infective endocarditis (caused by *Enterococcus* spp.). Other possible infections are infections of the auricle and endophthalmitis (caused by *Pseudomonas aeruginosa*). Mobile devices are often used in private and professional environments such as healthcare facilities.

An analysis of HCWs found that 58.3% of mobile phones were contaminated, with a higher contamination rate on the rear surfaces compared to the touchscreens [7]. The presence of bacteria on these devices poses a significant health risk, especially in healthcare settings where there is a greater chance of healthcare-associated infections (HAIs). Pathogens such as *Acinetobacter baumannii* and methicillin-resistant *Staphylococcus aureus* can cause serious infections in patients through the use of contaminated devices [2, 8].

Cross-transmission of mobile phones in the hospital setting is a global phenomenon. The study in Africa in Uganda, Zambia, Cameroon, and Nigeria had a 79%–96% contamination rate, and the most common bacteria isolated were coagulase-negative *Staphylococci*, *Staphylococcus aureus*, and *E. coli*, of which many were multidrug-resistant (MDR) [7, 9, 10]. Overall rate of contamination was 84.5% as deduced by a systematic review of 26 studies in Africa by Abubakar et al. [11].

Similarly, Asian studies also indicated extensive bacterial colonization in HCWs' phones, with the infection levels reaching even up to 72.6% in Pakistan, India, and Northeast India, even after frequent cleaning [12–14]. Research suggests applications of basic hygiene may be quite insufficient in curbing bacterial spread.

European contamination rates were even higher, with nearly every HCW's mobile phone being colonized by bacteria. A dramatic rise in clinically relevant pathogens was noted in one German study from 21.2% in 2012 to 39.8% in 2021, potentially linked to increased use of mobile devices in the context of the COVID-19 pandemic and suboptimal cleaning procedures [15]. An astonishing 94.5% of Turkish phones in one study were colonized by bacteria, with 52% of the *S. aureus* strains being methicillin-resistant and representing a concern for nosocomial transmission [16]. A UK study was even worse, with a report of up to six times as many bacteria being carried by a mobile phone as a toilet and yet owners do not clean them properly [17].

In the general population, the most prevalent pathogenic bacteria are *E. coli*, which can invade the gastrointestinal tract and the skin, leading to a range of infections. This underscores a critical concern for public health, the contamination of electronic devices. Given their widespread use and the tendency for them to come into contact with various surfaces, electronic devices can become reservoirs for these harmful microorganisms, posing a potential risk for transmission and illness within communities [3, 8]. Therefore, this study aimed to compare the bacterial contamination of electronic devices used by HCWs and NHCWs at Qassim University.

## 2. Materials and Methods

A comparative and experimental study with a triple factorial design to investigate bacterial contamination in electronic devices was conducted between September 2024 and November 2024. Swabs were taken from the devices of HCWs and NHCWs, males and females. The sociodemographic data of the study population are listed in Table 1. Before taking a swab, both hands were cleaned with an alcohol-based hand sanitizer, and gloves were worn. The screen of the device was swabbed with a sterile cotton swab soaked in a sterile saline solution. The swab from the device was quickly placed in sterile broth in a sterile container before being taken to the microbiology laboratory for less than 2 hours. A total of 200 samples were taken following Taha et al. [18]. To reflect usage frequency, samples were taken from 100 cell phones and 100 IPAD/PC. All participants indicated that they do not follow a proper hygienic protocol. Usage hours per day (±10 h/day) for cell phone participants and (±8 h/day) for IPAD/PC participants were assessed. Samples were cultured on blood agar (Oxoid, Cambridge, UK). The culture plates were incubated at 37°C for 48 h. Swap from unused electronic devices (control group) was also taken to establish baseline contamination.

After an incubation period of 48 h, the colonies growing on the plated media were subcultured to obtain a pure culture of each isolated colony type. The isolated microorganisms were identified using various microbiological identification tests, including biochemical and morphological characteristics: Gram stain, catalase, coagulase, oxidase, novobiocin, mannitol fermentation, and urease tests. Species-level identification was then confirmed by further testing using the VITEK 2 Compact System (bioMérieux, France). Antibiotic susceptibility tests were then performed using the disc diffusion method (Kirby–Bauer test). A standardized bacterial suspension was spread on the surface of a Mueller–Hinton agar (Oxoid, Cambridge, UK) plate. Paper discs impregnated with antibiotics were then placed on the agar surface. The antibiotics used were ampicillin, azithromycin, cefoxitin, ciprofloxacin, clindamycin, daptomycin, erythromycin, fosfomycin, fusidic acid, gentamicin, imipenem, levofloxacin, linezolid, moxifloxacin, mupirocin, nitrofurantoin, oxacillin, penicillin, rifampin, synercid, teicoplanin, tetracycline, and vancomycin. The plates were then incubated aerobically at 37°C for 24 hr to allow bacterial growth. The antibiotic zone diameter was measured with calipers and analyzed as per Clinical Laboratory and Standards Institute guidelines [19].

MDR is defined, as set out by the international consensus built by Magiorakos et al. [20], as the acquired inability to be susceptible to at least one antimicrobial drug from no less than three different classes of antimicrobials. Isolates were defined as MDR when this was met, based on resistance patterns determined by the disc diffusion method.

Ethical approval was not required for this research, as it was limited to laboratory experiments involving sampling from devices and did not involve human or animal subjects. Laboratory safety regulations and ethical standards were adhered to throughout the study period.

Independent sample *t*-tests were conducted to compare bacterial contamination levels based on gender, occupation (HCWs vs. NHCWs), and device type (cell phones vs. tablets/PCs), using SPSS Version 26. Levene's test for equality of variances was performed to evaluate the assumption of homogeneity. In cases where this assumption was violated, as indicated by significantly different variances, Welch's correction was utilized to ensure accurate results. Statistical significance was defined as *p* < 0.05, and all results were presented with corresponding *p* values, degrees of freedom (d*f*), 95% confidence intervals (CIs), and standard errors where applicable. Descriptive statistics were used to summarize the resistance patterns of the isolated bacterial strains. For each antibiotic-strain combination, the average resistance level (μg/mL) and standard deviation are reported. However, due to the absence of replicate measurements for each strain-antibiotic combination, inferential tests such as ANOVA could not be conducted on the resistance data, as the lack of within-group variance violates the fundamental assumptions of such analyses [21].

The bacteria isolated in this study were confirmed to be viable, meaning they were alive rather than dead, as demonstrated by their successful growth and colony formation in culture media after an incubation period. Live bacteria pose a greater risk than dead ones because they can multiply and easily spread infections to humans, especially in sensitive environments such as hospitals.

## 3. Results

The types and quantities of bacteria on electronic devices used by HCWs and NHCWs are reported in Table 2. *Staphylococcus epidermidis* was the most common, with 64 counts in HCW and 43 in NHCW, while *Acinetobacter lwoffii* had the least presence, with 6 in HCW and 0 in NHCW. HCW had a total of 212 bacterial colonies compared to 200 in the NHCW. Table 2 indicates that HCW showed more *S. intermedius* (9.5%), *Myroides* sp. (5.91%), and *Staphylococcus hominis* subsp. *novobiosepticus* (9%), whereas NHCW has higher levels of *Staphylococcus haemolyticus* (14.15%), *Rhodococcus equi* (13.2%), and *S. aureus* (13%). *Staphylococcus epidermidis* was more prevalent in HCW (29%) than in NHCW (20.28%), likely due to its association with hospital environments. *Acinetobacter lwoffii* and *S. haemolyticus* are found in NHCW (2% and 14.15%, respectively) but are absent in HCW. Control devices showed zero bacterial presence in all devices swabbed.

Table 3 shows the gender-based differences (*p* < 0.05) in bacterial prevalence. Male devices exhibited greater diversity, with significant differences (*p* < 0.05) between the sexes. They had higher colony numbers of *Staphylococcus sciuri* (29), *Rhodococcus equi* (29), and *S. aureus* (30). In contrast, females had higher rates of *S. epidermidis* (80 vs. 27 in males), *S. hominis* subsp. *novobiosepticus* (32), and *S. haemolyticus* (19). Notably, *Staphylococcus sciuri* and *A. lwoffii* were exclusive to the devices of males.

Table 4 highlights the device-based differences in bacterial prevalence. Cell phones showed higher contamination levels, with *S. sciuri*, *A. lwoffii*, *Myroides* sp., and *S. haemolyticus* exclusively found in them (100%). In addition, CNS bacteria, such as *S. epidermidis* (82.2%) and *S. hominis* subsp. *novobiosepticus* (86.5%), were mainly present on cell phones, suggesting their role in skin flora contamination. Contamination of iPads/PCs was low, with *S. aureus* (52.9%) and gram-positive *bacilli* (34.8%) being the most common bacteria, but still less than on cell phones. The descriptive analysis of antibiotic resistance revealed substantial variability in resistance patterns across the six gram-positive bacterial strains isolated from electronic devices (Table 5). *Staphylococcus haemolyticus* and *S. intermedius* exhibited notably high resistance to mupirocin (55.1 μg and 64.3 μg, respectively), while elevated resistance to nitrofurantoin was also observed in *S. intermedius* (16.1 μg). In contrast, resistance levels to antibiotics such as clindamycin, rifampin, and ciprofloxacin were generally low across most isolates. Several strains, including *S. epidermidis* and *S. hominis* subsp. *hominis*, demonstrated moderate MDR, particularly to fosfomycin and azithromycin. These findings underscore the potential for device-mediated transmission of resistant organisms, especially in healthcare-associated settings.

## 4. Discussion

This study has shown that the electronic devices used by students, employees, and medical staff at Qassim University harbor various microorganisms. The antimicrobial resistance profiles of these microbes may influence the level of contamination of these devices. Most of the devices exhibited polymicrobial growth, with 86% showing this characteristic. Coagulase-negative bacteria were the predominant species found to a significant extent. Although coagulase-negative bacteria are commonly found on human skin, they can pose a serious risk, especially in immunocompromised individuals [22]. In addition, Bodena et al. [23] identified coagulase-negative bacteria as the primary isolates, accounting for 58.8% of cases, while Koscova et al. [24] reported an even higher rate of 76%. Among the isolates from HCWs, *S. aureus* was found to be present in 13% of the samples, which was significantly lower than the 24.5% observed in a study by Mushabati et al. [9]. On the other hand, *Acinetobacter* species occurred at a similar frequency of 2% in both studies. These results show that the patterns of bacterial contamination of medical staff VDUs are similar in the different studies. However, the differences in the prevalence of certain bacteria indicate different environmental factors, sampling techniques, and healthcare facilities.

In this study, different types of bacteria were detected, with *S. epidermidis* being the most prevalent. In contrast, a study conducted by Tambe and Pai [25], in India, found a different bacterial profile, with *S. aureus* being the most common bacterium found on device screens. The discrepancies in bacterial types between the two studies can be attributed to factors such as environmental conditions, healthcare practices, and types of devices used by HCWs.

Significant gender differences were observed in the types and prevalence of bacteria isolated from the screens of the devices. In particular, *S. epidermidis* was more prevalent in women's devices (39.6%) than in men's devices (12.8%). The prevalence of *S. aureus* was higher in men's devices (14.2%) than in women's devices (1.98%). A similar study by Elmanama et al. [26] conducted in Palestine showed a different distribution of bacteria in women and men. *Staphylococcus aureus* was prevalent in male devices in 27% of the samples and 11% of the female samples.

Mobile phones, in particular, have a higher bacterial load than other devices. These results suggest that mobile phones are a large reservoir for bacterial contamination, probably due to frequent handling and exposure to different environments. According to a review by Olsen et al. [8], bacterial species isolated from mobile phones have been studied in both healthcare environments and wider communities. The study found that *S. aureus* and coagulase-negative *staphylococci* were the microorganisms most frequently encountered on mobile devices in the majority of studies. Some studies have reported that the diverse microbial life found on these devices is very similar to that found in everyday environments [8].

In this study, the bacteria that were isolated exhibited MDR, suggesting significant concerns regarding antimicrobial efficacy. In a related investigation conducted by Banawas et al. [27], MDR bacteria associated with the cell phones of healthcare professionals in selected hospitals in Saudi Arabia were examined. The results indicated that a majority of coagulase-negative *staphylococci* demonstrated resistance to benzylpenicillin, erythromycin, and rifampicin. Furthermore, eight isolates were found to be resistant to oxacillin, specifically including *S. epidermidis*, *S. hominis*, and *S. warneri*. It is also worth noting that *A. otitis*, recognized for its role in causing acute otitis media, presented with MDR as well. Importantly, one isolate of hetero-vancomycin intermediate-resistant *S. aureus* exhibited resistance to commonly prescribed antibiotics for managing skin infections. These findings highlight the persistent challenge of combating antibiotic resistance in clinical settings. However, the study has some limitations, including the fact that samples were collected only from one geographic location, there are no longitudinal data available, and there may be potential sampling bias. It also overlooked factors such as personal hygiene, device usage rate, and environmental exposures, which may influence contamination levels. These uncontrolled variables might partly explain the differences observed concerning gender and occupation.

Understanding the various types of bacteria and their presence on devices can assist in implementing effective hygiene measures. It can also promote the development of antimicrobial coatings or cleaning agents specifically designed for these devices.

Mobile phones used by both HCWs and NHCWs can carry pathogenic and drug-resistant bacteria, posing serious public health risks. For example, HCWs' devices show contamination rates as high as 93% in intensive care units. Notably, MDR bacteria have been found on these phones, raising concerns about cross-transmission between workers and patients, potentially leading to outbreaks [9].

Guidelines for preventing infections are being updated to include mobile phone decontamination, particularly in healthcare settings. Traditional infection control has focused on hand hygiene and environmental cleanliness, often overlooking mobile phones, which can harbor high contamination levels. Hospitals are now adding mobile phone cleaning to their protocols, especially in intensive care units, using 70% alcohol wipes and UV-C disinfection tools that can eliminate over 99% of bacteria without damaging the devices. There is also growing recognition of mobile phones' role in antimicrobial stewardship programs, particularly regarding MRSA, with research highlighting antibiotic-resistant bacteria found on phones. Despite this, compliance with cleaning protocols is low; a study in Pakistan found 72.6% of mobile phones contaminated, even when users believed they had cleaned them. This underscores the need for regular cleaning protocols, staff training, and incorporating device hygiene into hospital checklists.

## 5. Conclusion

Electronic devices were found to harbor live, MDR gram-positive bacteria, increasing the risk of infection transmission, particularly in hospital environments. Contamination was greater on mobile phones than on tablets/PCs and was higher among devices used by male participants and HCWs.

These findings emphasize the necessity of integrating routine device disinfection and hygiene education into infection-control protocols. Observations for future research should be prolonged to enhance preventive strategies.

## Figures and Tables

**Table 1:** Sociodemographic information for the study population.

Sociodemographic characteristics	Number
Gender	
Female	100 (50%)
Male	100 (50%)
Occupation	
University students	70
University employers	30
Medical doctors	20
Nurses	40
Quality staff in the hospital	40

**Table 2:** *T*-test results comparing bacterial contamination levels by occupation.

The type of bacteria isolated	HCW (*n*)	%	NHCW (*n*)	%	*t*	d*f*	*p* value	95% CI	SE
*Staphylococcus sciuri*	19	9.0	10	5.0	2.01	198	0.045	[0.02, 0.18]	0.044
*Acinetobacter lwoffii*	6	2.8	0	0.0	2.59	198	0.011	[0.01, 0.10]	0.038
*Staphylococcus aureus*	29	13.7	5	2.5	5.74	198	< 0.001	[0.15, 0.33]	0.049
*Myroides* sp.	10	4.7	13	6.5	−0.68	198	0.498	[−0.12, 0.06]	0.042
*Staphylococcus hominis* subsp hominis	9	4.2	9	4.5	0.0	198	1.0	[−0.08, 0.08]	0.041
*Staphylococcus haemolyticus*	30	14.2	0	0.0	7.91	198	< 0.001	[0.20, 0.38]	0.047
*Staphylococcus epidermidis*	43	20.3	64	32.0	−2.55	198	0.012	[−0.21, −0.03]	0.047
*Staphylococcus intermedius*	5	2.4	21	10.5	−3.76	198	< 0.001	[−0.20, −0.06]	0.037
*Rhodococcus equi*	28	13.2	16	8.0	2.02	198	0.045	[0.01, 0.20]	0.046
Gram-positive bacilli	10	4.7	13	6.5	−0.64	198	0.522	[−0.10, 0.05]	0.041
*Micrococcus*	15	7.1	20	10.0	−0.95	198	0.343	[−0.09, 0.03]	0.038
*Staphylococcus hominis* subsp novobiosepticus	8	3.8	29	14.5	−4.36	198	< 0.001	[−0.22, −0.09]	0.047

*Note:* Percentages represent the proportion of participants with bacterial contamination as per the occupational group. Levene's test for equality of variances guided whether the standard *t*-test or Welch's correction was used. Significance was considered at *p* < 0.05.

**Table 3:** *T*-test results comparing bacterial contamination levels by gender.

The type of bacteria isolated	Male (*n*)	%	Female (*n*)	%	*t*	df	*p* value	95% CI	SE
*Staphylococcus sciuri*	100	29.0%	100	0.0%	6.359	99	< 0.001	[0.1995, 0.3805]	0.0456
*Acinetobacter lwoffii*	100	6.0%	100	0.0%	2.514	99	0.014	[0.0126, 0.1074]	0.0239
*Staphylococcus aureus*	100	19.0%	100	15.0%	0.750	196	0.454	[−0.0651, 0.1451]	0.0533
Myroides sp.	100	8.0%	100	15.0%	−1.553	184	0.122	[−0.1589, 0.0189]	0.0451
*Staphylococcus hominis* subsp hominis	100	15.0%	100	3.0%	3.017	142	0.003	[0.0414, 0.1986]	0.0398
*Staphylococcus haemolyticus*	100	11.0%	100	19.0%	−1.586	188	0.114	[−0.1795, 0.0195]	0.0504
*Staphylococcus epidermidis*	100	27.0%	100	80.0%	−8.825	195	< 0.001	[−0.6484, −0.4116]	0.0601
*Staphylococcus intermedius*	100	12.0%	100	14.0%	−0.419	197	0.676	[−0.1142, 0.0742]	0.0478
*Rhodococcus equi*	100	29.0%	100	15.0%	2.412	187	0.017	[0.0255, 0.2545]	0.0580
Gram-positive bacilli	100	13.0%	100	10.0%	0.662	195	0.509	[−0.0593, 0.1193]	0.0453
*Micrococcus*	100	19.0%	100	16.0%	0.556	197	0.579	[−0.0764, 0.1364]	0.0540
*Staphylococcus hominis* subsp novobiosepticus	100	5.0%	100	32.0%	−5.218	140	< 0.001	[−0.3723, −0.1677]	0.0518

*Note:* Percentages represent the proportion of participants with bacterial contamination as per the gender group. Levene's test for equality of variances was applied to assess homogeneity. Welch's correction was used when variances were unequal (as indicated by unequal d*f*). Statistical significance was defined as *p* < 0.05.

**Table 4:** *T*-test results comparing bacterial contamination levels by device type.

The type of bacteria isolated	IPAD/PC (*n*)	%	Cell phone (*n*)	%	*t*	d*f*	*p* value	95% CI	SE
*Staphylococcus sciuri*	0	0	29	8.7	9.12	198	< 0.001	[0.35, 0.52]	0.049
*Acinetobacter lwoffii*	0	0	6	1.8	2.59	198	0.011	[0.01, 0.10]	0.038
*Staphylococcus aureus*	18	22.8	16	4.8	−0.4	198	0.689	[−0.12, 0.08]	0.05
Myroides sp.	0	0	23	6.9	8.87	198	< 0.001	[0.34, 0.51]	0.047
*Staphylococcus hominis* subsp hominis	5	6.3	13	3.9	2.68	198	0.008	[0.03, 0.16]	0.041
*Staphylococcus haemolyticus*	0	0	30	9	7.91	198	< 0.001	[0.20, 0.38]	0.047
*Staphylococcus epidermidis*	19	24	88	26.4	2.05	198	0.042	[0.01, 0.18]	0.046
*Staphylococcus intermedius*	6	7.6	20	6	2.13	198	0.034	[0.01, 0.16]	0.044
*Rhodococcus equi*	10	12.7	34	10.2	2.17	198	0.031	[0.01, 0.18]	0.045
Gram-positive bacilli	8	10.1	15	4.5	1.89	198	0.06	[−0.01, 0.21]	0.049
*Micrococcus*	8	10.1	27	8.1	3.12	198	0.002	[0.03, 0.13]	0.041
*Staphylococcus hominis* subsp novobiosepticus	5	6.3	32	9.6	4.41	198	< 0.001	[0.12, 0.28]	0.048

*Note:* Contamination percentages are reported as per device category. Welch's correction was applied for variables violating the assumption of equal variances, based on Levene's test. Results with *p* < 0.05 were considered statistically significant.

**Table 5:** Mean resistance values (μg) ± standard deviations of antibiotics against bacterial strains isolated from electronic devices.

Antibiotic	*Staphylococcus sciuri*	*Staphylococcus aureus*	*Staphylococcus hominis subsp. hominis*	*Staphylococcus haemolyticus*	*Staphylococcus epidermidis*	*Staphylococcus intermedius*
Ampicillin	0.25 ± 0.82	5.81 ± 0.43	3.46 ± 0.77	1.72 ± 0.08	5.51 ± 0.70	0.06 ± 0.47
Azithromycin	1.99 ± 0.79	1.45 ± 1.10	1.73 ± 0.55	0.86 ± 0.21	2.75 ± 0.90	1.00 ± 0.66
Cefoxitin screen	3.98 ± 0.29	2.90 ± 1.48	3.46 ± 0.02	0.86 ± 0.57	2.75 ± 0.56	1.00 ± 0.78
Ciprofloxacin	0.99 ± 0.25	1.45 ± 0.51	0.87 ± 0.66	0.22 ± 0.04	0.69 ± 0.61	0.25 ± 0.20
Clindamycin	1.99 ± 1.28	0.18 ± 0.74	0.22 ± 0.93	0.43 ± 0.86	1.38 ± 1.30	0.13 ± 1.49
Daptomycin	0.99 ± 1.29	0.73 ± 0.48	0.87 ± 0.73	0.86 ± 0.08	0.69 ± 0.90	0.25 ± 0.80
Erythromycin	0.50 ± 0.76	0.36 ± 0.44	0.43 ± 1.22	0.86 ± 1.44	2.75 ± 1.47	1.00 ± 0.92
Fosfomycin	31.80 ± 0.83	23.20 ± 0.50	27.70 ± 0.31	6.89 ± 1.50	22.00 ± 0.08	8.03 ± 0.98
Fusidic acid	1.99 ± 1.31	11.60 ± 1.02	1.73 ± 0.15	3.44 ± 0.23	11.00 ± 0.84	0.50 ± 0.42
Gentamicin	0.99 ± 0.42	0.73 ± 1.25	0.87 ± 0.29	1.72 ± 0.85	0.69 ± 0.48	2.01 ± 0.26
Imipenem	3.98 ± 1.12	2.90 ± 1.23	3.46 ± 0.91	0.86 ± 1.40	2.75 ± 0.04	1.00 ± 0.17
Levofloxacin	0.99 ± 0.39	2.90 ± 0.77	0.87 ± 1.17	0.22 ± 0.20	2.75 ± 0.16	0.50 ± 1.07
Linezolid	1.99 ± 0.92	2.90 ± 0.41	1.73 ± 1.10	0.86 ± 0.84	0.69 ± 0.69	0.50 ± 0.61
Moxifloxacin	0.50 ± 1.17	0.73 ± 1.22	0.43 ± 0.15	0.11 ± 0.80	1.38 ± 0.48	0.25 ± 1.24
Mupirocin	3.98 ± 1.08	2.90 ± 1.43	3.46 ± 1.03	55.10 ± 0.78	0.34 ± 0.13	64.30 ± 0.04
Nitrofurantoin	31.80 ± 0.49	23.20 ± 1.34	27.70 ± 0.67	13.80 ± 0.54	0.34 ± 0.12	16.10 ± 0.28
Oxacillin	0.25 ± 1.11	1.45 ± 1.43	0.22 ± 0.71	0.05 ± 0.41	22.00 ± 1.14	0.50 ± 0.08
Penicillin	0.03 ± 0.60	5.81 ± 1.31	6.93 ± 1.45	1.72 ± 0.27	0.17 ± 1.44	0.03 ± 0.37
Rifampin	0.99 ± 1.23	0.73 ± 1.03	0.87 ± 0.77	0.43 ± 1.01	5.51 ± 0.37	0.25 ± 1.24
Synercid	0.99 ± 1.21	0.73 ± 1.20	0.87 ± 1.09	0.43 ± 0.17	0.69 ± 0.98	0.25 ± 0.40
Teicoplanin	3.98 ± 0.63	2.90 ± 0.01	6.93 ± 0.86	3.44 ± 0.68	0.69 ± 0.75	1.00 ± 0.66
Tetracycline	3.98 ± 1.28	2.90 ± 0.21	3.46 ± 0.99	1.72 ± 0.90	11.00 ± 1.41	1.00 ± 0.77
Vancomycin	0.99 ± 1.47	1.45 ± 1.09	1.73 ± 1.10	3.44 ± 1.44	1.38 ± 1.12	0.13 ± 1.35

*Note:* Values are presented as mean resistance levels in μg/mL ± standard deviation for each bacterial strain–antibiotic combination. Resistance measurements are based on disc diffusion assay interpretations.

## Data Availability

The authors declare that the data supporting the findings of this study are included in this article. If any raw data files are needed in another format, they are available from the corresponding author upon reasonable request.
